# Supplementary material for: Evaluation of Four Strategies for SARS-CoV-2 Detection: Characteristics and Prospects
Source: Microbiol Spectr. 2022 Oct 26;10(6):e02143-22. doi: 10.1128/spectrum.02143-22 (PMC9769534; doi:10.1128/spectrum.02143-22)
Supplement: Supplemental file 1 — Supplemental material. Download spectrum.02143-22-s0001.pdf, PDF file, 0.1 MB [file spectrum.02143-22-s0001.pdf]

Supplementary Material File 1

Table S1 The interpretations of the first test results and retest results of the five rRT-PCR tests

**Study kits**	**Targrt gene(s)**	**Positive result**		**Retest**	**Retest positive result**	
		**Ct**	**Result interpretation**		**Ct**	**Retset result interpretation**
Daan	ORF1ab, N	≤40	ORF1ab (+) and N (+)	ORF1ab (+) or N (+)	≤40	ORF1ab (+) or N (+)
BioGerm	ORF1ab, N	≤40	ORF1ab (+) and N (+)	ORF1ab (+) or N (+)	≤40	ORF1ab (+) or N (+)
Liferiver	ORF1ab, N, E	≤43	ORF1ab (+) and N (+) and E (+); ORF1ab (+) and N (+) or E (-); ORF1ab (+) and N (-) or E (+); ORF1ab (-) and N (+) and E (+)	ORF1ab (+) and N (-) and E (-); ORF1ab (-) and N (+) and E (-)	≤43	ORF1ab (+) or N (+) or E (+)
Easydiagnosis	ORF1ab, N	< 38	ORF1ab (+) and N (+)	ORF1ab (+) or N (+); ORF1ab or N (38≤ Ct < 40)	<40	ORF1ab (+) and N (+)
Sansure	ORF1ab, N	≤40	ORF1ab (+) or N (+)	/	/	/

Ct: cycle threshold, the thermal cycle number at which the fluorescent signal exceeds that of the background and thus passes the threshold for positivity.

Supplementary Material File 2

For the single sampling tests and 20-in-1 pooling tests, 50 μL cell culture supernatants were added to 3 mL and 12 mL sample preservation solution. The concentration equivalent to the 50 μL sample was the lowest concentration to achieve 100% detection rates of the 50 μL original sample before being added to the sample preservation solution. The lowest concentration at 100% detection rates of the diluted sample was 1/61 (single sampling tests, 50 μL in 3050 μL) or 1/241 (20-in-1 pooling tests, 50 μL in 12050 μL) of the 50 μL original concentration. Assuming that the concentration equivalent to the 50 μL sample for the single sampling tests using the Daan kit was 6.25×10^4 copies/mL, then the lowest concentration at 100% detection rates was 6.25×10^4 divided by 61, namely 1.02×10^3 copies/mL.

For the point-of-care test and rapid antigen tests, the multiples varied according to the amount of extraction solution of different kits. Taking the antigen test BGI as an example, the volume of extraction solution was 400 μL and the lowest concentration at 100% detection rates was 1/9 (50 μL in 450 μL) of the concentration equivalent to the 50 μL sample. Assuming that the concentration equivalent to the 50 μL sample for the BGI test was 3.12×10^6 copies/mL, then the lowest concentration at 100% detection rates was 3.12×10^6 divided by 9, namely 3.47×10^5 copies/mL.
